# Impact of paravertebral block on perioperative neurocognitive disorder: a systematic review and meta-analysis of randomized controlled trials

**DOI:** 10.3389/fnagi.2023.1237001

**Published:** 2023-10-03

**Authors:** Lu Wang, Fei Wang, Wanli Kang, Guangkuo Gao, Tao Liu, Bin Chen, Wei Liu

**Affiliations:** ^1^Beijing Tuberculosis and Thoracic Tumor Research Institute, Beijing Chest Hospital Capital Medical University, Beijing, China; ^2^Department of Anesthesiology, Beijing Chest Hospital, Capital Medical University, Beijing, China; ^3^Department for Disease Prevention and Control, Beijing Chest Hospital, Capital Medical University, Beijing, China

**Keywords:** paravertebral block, surgery, perioperative neurocognitive disorder, postoperative delirium, delayed neurocognitive recovery, postoperative pain

## Abstract

**Objective:**

To investigate whether paravertebral block reduces postoperative delirium (POD)/delayed neurocognitive recovery (DNR) in adults after major surgery with general anesthesia.

**Methods:**

For this systematic review and meta-analysis, we searched online databases PubMed, EMBASE, CENTRAL, and Web of Science till March 19th, 2023 to examine studies which use paravertebral block (PVB) for perioperative neurocognitive disorder. Primary and secondary outcomes were identified for the incidence of perioperative neurocognitive disorder. We did not restrict the follow-up duration of the included studies. Statistical analysis was performed to calculate mean difference (MD), Odd ratios (OR) and CI between RCTs. The quality of the evidence was assessed with the Cochrane risk of bias tool. The registration number of the study in PROSPERO is CRD42023409502. PROSPERO is an international database of prospectively registered systematic reviews. Registration provides transparency in the review process and it helps counter publication bias.

**Results:**

Total 1,225 patients from 9 RCTs were analyzed. The incidence of POD [Odds Ratio (OR) = 0.48, 95% CI 0.32, 0.72; *p* = 0.0004; *I*^2^ = 0%] and DNR [OR = 0.32, 95% CI 0.13, 0.80; *p* = 0.01; *I*^2^ = 0%] were significantly reduced in PVB group. The analysis showed no significant differences in postoperative MMSE scores [MD = 0.50, 95% CI −2.14, 3.15; *p* = 0.71; *I*^2^ = 98%]. Paravertebral block analgesia reduces pain scores and/or opioid use after surgery. Additionally, blood pressure was significantly lower in the PVB group, intraoperatively [MD = −15.50, 95% CI −20.71, −10.28; *p* < 0.001; *I*^2^ = 12%] and postoperatively [MD = −5.34, 95% CI −10.65, −0.03 *p* = 0.05; *I*^2^ = 36%]. Finally, PVB group had significantly shorter hospital stays [MD = −0.86, 95% CI −1.13, −0.59; *p* < 0.001; *I*^2^ = 0%].

**Conclusion:**

Paravertebral block analgesia may prevent perioperative POD/DNR in patients undergoing major surgery. Further research with large sample sizes is required to confirm its effectiveness.

## Introduction

Previous research indicates that a significant proportion of individuals, approximately 30–50% experience perioperative neurocognitive disorder (PND) (Monk et al., 2008; Mahr et al., 2021). It includes postoperative delirium (POD), delayed neurocognitive recovery (DNR) and postoperative cognitive dysfunction (POCD) (Evered et al., 2018). PND is the most common (Chinnappa-Quinn et al., 2020) complication in patients after prolonged anesthesia and surgery, and has been linked with an increased risk of accelerated age-related cognitive decline, dementia, and mortality (Monk et al., 2008). Additionally, PND can have negatively effect on patients’ long-term cognitive function and quality of life (Needham et al., 2017). The incidence of POD and POCD varies according to patient population, types of surgery, and anesthetic management (Jin Z. et al., 2020; Wang et al., 2023).

Postoperative pain management is an important factor in major surgical procedures that should not be overlooked. Paravertebral block (PVB) has been a widely used regional anesthesia technique for postoperative analgesia in a variety of surgeries, particularly for thoracic and upper abdominal surgeries. This technique offers adequate pain relief and help reduce the need for opioids by decreasing morphine consumption (Gürkan et al., 2020; Liu et al., 2022). Additionally, PVB also found as effective as epidural analgesia in managing postoperative pain and to cause fewer side effects (Davies et al., 2006; Zeng et al., 2022).

Literature has provided significant evidence in favor of multimodal analgesic techniques such as a combination of regional anaesthetic blockade and systemic analgesia in major surgeries (Yoshimura et al., 2022). However, despite extensive research on analgesic techniques, there is limited information on PVBs effectiveness in improving patient’s outcomes by reducing the occurrence of PND. The purpose of this systematic review and meta-analysis is to explore the effect of PVB on the frequency of PND. The authors have extensively reviewed the available literature and found that no meta-analysis comprehensively inspected the effectiveness of PVB in reducing PND occurrence.

## Methods

The study was conducted following the guidelines set by the Preferred Reporting Items for Systematic Reviews and Meta Analyses (PRISMA) (Moher et al., 2009). We registered the protocol for this systematic review on the International Prospective Register of Systematic Reviews (PROSPERO) under the registration number: CRD42023409502.

### Study selection criteria

Inclusion Criteria required the following: (1) randomized controlled trials; (2) patients who underwent major surgery and received PVB during the perioperative period; (3) patient age ≥ 18 years; (4) patients who have faced postoperative PND.

Exclusion Criteria included: (1) case reports, observational studies, retrospective studies, review articles, or comments; (2) incomplete publications, only including abstracts or protocols.

### Search strategy

We search for trials that meet our requirements from online databases such as PubMed, EMBASE, CENTRAL, and Web of Science, with no limitation on year of publication, language, journal, or region from creation of the database to March 19, 2023. The following key words such as “postoperative cognitive dysfunction,” “delayed neurocognitive recovery,” “postoperative delirium” and “paravertebral block” were used as query to gather specific information: The full electronic search strategies used are shown in the Supplemental Digital Content (Appendix 1).

### Study selection

Study selection was based on independent screening of the titles and abstracts by two investigators (LW and FW). Subsequently, the selected studies were examined for their full text. The eligibility of the selected studies was determined by the same two reviewers separately and in duplicate. In case of discrepancies regarding studies’ eligibility, a third author (WL) was consulted for clarity. Observational studies, case reports, conference abstracts, reviews, meta-analysis, and case series were not considered.

### Data extraction

Two researchers independently screened the literature, extracted the data, and evaluated the methodological quality of the studies identified. The information was organized in a spreadsheet, including authors name, publication year, study design, number of participants, type of surgery, follow-up duration, tools used for cognitive assessment, participants’ age, cognitive outcomes and test scores, type of nerve block, local anesthetics used in PVB, and postoperative PCA medication and setting. The median and IQR were utilized to determine mean and standard deviation of the continuous data (Shi et al., 2020). Numerical values of graphs were obtained using Web Plot Digitizer, and combine mean and SD from multiple groups was calculated using a calculation tool provided by Combine Mean SD.^1^

### Literature screening, data extraction, and quality assessment

Two reviewers independently assessed the risk of bias of nine using the revised Cochrane Risk of Bias Assessment Tool (Sterne et al., 2019). The evaluation included random sequence generation, allocation concealment, blinding of investigators and participants, blinding of outcome assessment, incomplete outcome data, selective reporting, and other biases. Disputes between the two researchers were resolved through discussion or by consulting a third party to reach a consensus. The publication bias was detected by the Egger method.

### Outcome measurement

The primary outcome was the incidence of postoperative POD or DNR. Secondary outcomes included postoperative MMSE scores (cognitive tests), preoperative and postoperative S-100β levels, resting pain scores at 24 and 48 h postoperatively (using VAS scores or NRS scores), postoperative opioid consumption, mean arterial pressure levels, and duration of hospitalization.

### Statistical analysis

Meta-analyses were performed with the assistance of Review Manager software (RevMan version 5.4.1) and Egger method in Stata 17.0 was employed to identify publication bias. Binary variables of outcomes including postoperative delirium and postoperative cognitive dysfunction were reported as ORs with 95% CI. Continuous variables including MMSE score, NRS score, opioid consumption, blood pressure, biomarker levels and hospitalization duration was reported as mean difference (MD) with 95% CI.

The sensitivity analysis was conducted by using leave-one-out method to evaluate whether a single study can affect the pooled effect sizes. The level of heterogeneity of pooled-effect estimates was examined using *I*^2^ statistics. Heterogeneity among the studies was categorized as high (*I*^2^ = 76%–100%), moderate (*I*^2^ = 26%–75%), or low (*I*^2^ = 0%–25%). A random-effect model was used in cases where *I*^2^ > 50%, suggesting the existence of high heterogeneity, whereas fixed model was employed if *I*^2^ ≤ 50%. Statistical significance was set at *p* < 0.05.

## Results

### Study selection

**Figure 1:**
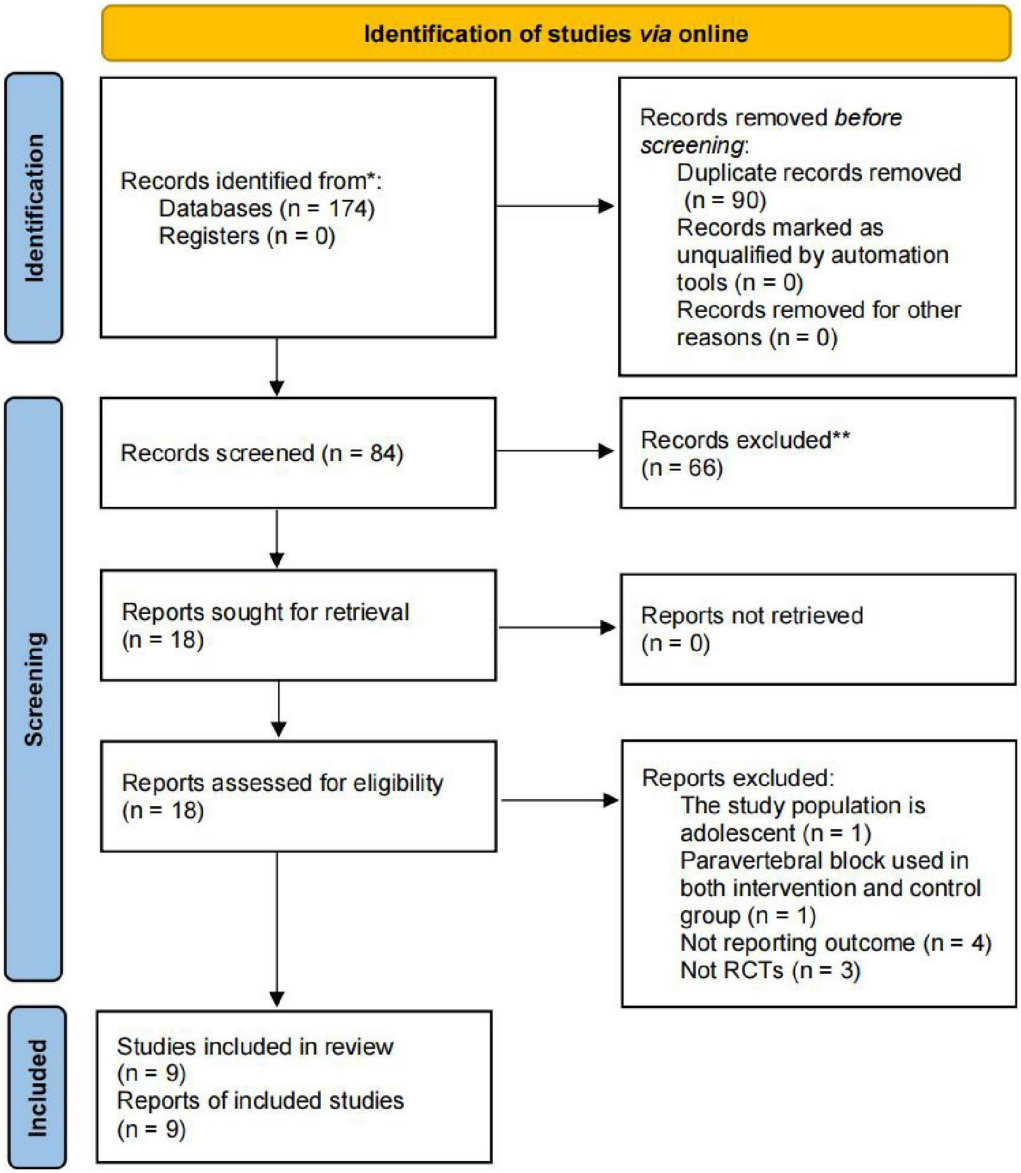
Flow chart illustrating the study design and selection criteria for RCTs. *Consider, if feasible to do so, reporting the number of records identified from each database or register searched (rather than the total number across all databases/registers). **If automation tools were used, indicate how many records were excluded by a human and how many were excluded by automation tools.

Initially, 174 articles were retrieved from the online database. After eliminating duplicates, 148 titles and abstracts were reviewed (Prisma flow chart). Out of these, 90 documents were excluded because of duplication, and 66 were excluded based on the relevance of their titles and abstracts. In the end, 9 RCTs were selected for final analysis (Figure 1).

Table 1 includes details about the RCTs, among them six RCTs (Xie et al., 2019; Wei et al., 2020, 2022; Chen et al., 2022; Heng et al., 2022; Dongjie et al., 2023) enrolled patients who underwent thoracoscopic surgery, one (Jin L. et al., 2020) enrolled patients who underwent esophagectomy, one (Shang et al., 2022) enrolled patients who underwent gastrectomy, and one (Strike et al., 2019) enrolled patients who underwent Transapical Aortic Valve Replacement. Five RCTs compared the incidence of POD between PVB and non-PVB groups (Strike et al., 2019; Jin L. et al., 2020; Heng et al., 2022; Wei et al., 2022; Dongjie et al., 2023), while other five RCTs, compared the incidence of DNR (Xie et al., 2019; Wei et al., 2020; Chen et al., 2022; Heng et al., 2022; Shang et al., 2022). Only one study (Chen et al., 2022) reported the incidence of POCD (Table 2). Among the RCTs, five (Strike et al., 2019; Jin L. et al., 2020; Wei et al., 2020; Heng et al., 2022) used continuous paravertebral analgesia post-operation, and four used the same intravenous analgesia PCA settings between PVB and non-PVB groups (Xie et al., 2019; Chen et al., 2022; Shang et al., 2022; Dongjie et al., 2023). It’s worth noting that, except one study Chen et al., all participants elderly.

**Table 1:** Patients details and surgery type of included RCTs.

Author and year	Design	Total patients	Types of surgery	Follow-up duration	Measurement scale	Mean age (years)	Sex, male	Hypertension	Diabetes mellitus	Education background (years)	Outcomes assessed
Strike et al. (2019)	2-arm RCT	44	Transapical aortic valve replacement	48 h	CAM-ICU	82.3 ± 6.1	19 (43.2%)	44 (100%)	11 (25%)	N	POD
						81.7 ± 5.7					
Xie et al. (2019)	3-arm RCT	77	Thoracoscopic lobectomy	7d	MMSE, digital symbol test, digital breadth test, trail connection test A	75.13 ± 5.60	N	N	N	N	DNR
						76.63 ± 4.60					
Jin Z. et al. (2020) and Jin L. et al. (2020)	2-arm RCT	167	Elective esophagectomy for stage III-IV esophageal cancer	4d	CAM	70.8 ± 5.2	77 (85.6%)	59 (65.6%)	35 (38.9%)	N	POD
						71.4 ± 5.6					
Wei et al. (2020)	3-arm RCT	90	Thoracoscopic lobectomy	7d	MMSE	74.7 ± 5.1	48 (53.3%)	N	N	8.57 ± 3.91	DNR
						73.5 ± 4.5					
Heng et al. (2022)	2-arm RCT	128	Thoracoscopic-assisted radical resection of NSCLC (nonsmall cell lung cancer)	4d	Nu-DESC	70.3 ± 5.5	70 (54.7%)	N	N	N	POD, DNR
						69.7 ± 6.1					
Wei et al. (2022)	2-arm RCT	338	VATS lobectomy	7d	3D-CAM	76.2 ± 6.3	163 (48.2%)	158 (46.7%)	75 (22.2%)	N	POD
						73.5 ± 7.1					
Shang et al. (2022)	2-arm RCT	60	Radical gastrectomy surgery	3d	MMSE	66 (65–78)	40 (66.7%)	40 (66.7%)	33 (55.0%)	4.10 ± 2.90	DNR
						65.5 (64.5–75)					
Chen et al. (2022)	3-arm RCT	110	Elective thoracoscopic radical lung cancer surgery	1d, 3 m	MMSE	58.81 ± 5.58	51 (46.4%)	N	N	N	DNR, POCD
						56.46 ± 6.07					
Dongjie et al. (2023)	2-arm RCT	211	Thoracoscopic lobectomy	5d	Nu-DESC	68 ± 5	126 (60.6%)	113 (54.3%)	78 (37.5%)	N	POD
						69 ± 4					

Nu-DESC, The Nursing Delirium Screening Scale; MMSE, Mini-Mental State Examination; CAM, Confusion Assessment Method; 3D-CAM, 3-min Diagnostic Interview for CAM; N, Not available.

**Table 2:** Details of intervention drugs used in the selected RCTs.

Author and year	Sample size (intervention vs. control arm)	Local anesthetics	PCA
Strike et al. (2019)	Paravertebral group = 22	5–8 mL of 0.5% ropivacaine or 0.25% bupivacaine	0.2% ropivacaine or 0.125% bupivacaine (5–10 mL/h) combined with supplemental analgesia
	PCA group = 22	/	supplemental analgesia (hydromorphone; NSAIDS or ketorolac; acetaminophen)
Xie et al. (2019)	TPVB-GA group = 39	20 mL of 0.25% ropivacaine	100 μg of sufentanil +200 mg of flurbiprofen axetil
	GA group = 38	/	
Jin Z. et al. (2020) and Jin L. et al. (2020)	PVB group = 84	15–20 mL 0.375% ropivacaine and 10 mg sufentanil	0.375% ropivacaine and 10 mg of sufentanil were injected through the catheter every 6 h
	PCA group = 83	/	sufentanil 0.05 mg/kg/h and a bolus dose with sufentanil 0.03 mg/kg
Wei et al. (2020)	TA group = 30	0.5% ropivacaine (5 mg/mL)	0.25% ropivacaine(Loading dose 0.5 mL, rate 2 mL/min)
	GA group = 30	/	2 μg/kg sufentanil(Loading dose 0.5 mL, rate 2 mL/min)
Heng et al. (2022)	TPVB group = 64	20 mL 0.5% ropivacaine	0.2% ropivacaine (Loading dose 2 mL, rate 2 mL/min)
	PCA group = 64	/	sufentanil 2–3 μg/kg
Wei et al. (2022)	PBA group = 170	5 mL of 1% lidocaine	0.2% ropivacaine(rate 2 mL/h)
	PIA group = 168	/	2 μg/kg sufentanil(rate 2 mL/h)
Shang et al. (2022)	PVB group = 30	20 mL of 0.375% ropivacaine	100 μg sufentanil
	C group = 30	/	
Chen et al. (2022)	TP group = 36	0.375% (20 mL) ropivacaine	0.04 μg/kg sufentanil(rate 2 mL/h)
	C group = 37	/	
Dongjie et al. (2023)	T group = 103	20 mL 0.375% ropivacaine	dexocine 1 mg/ kg + ketorolac ambutritol 3 mg/kg
	C group = 105	/	

Nu-DESC, The Nursing Delirium Screening Scale; MMSE, Mini-Mental State Examination; CAM, Confusion Assessment Method; 3D-CAM, 3-min Diagnostic Interview for CAM.

**Figure 2:**
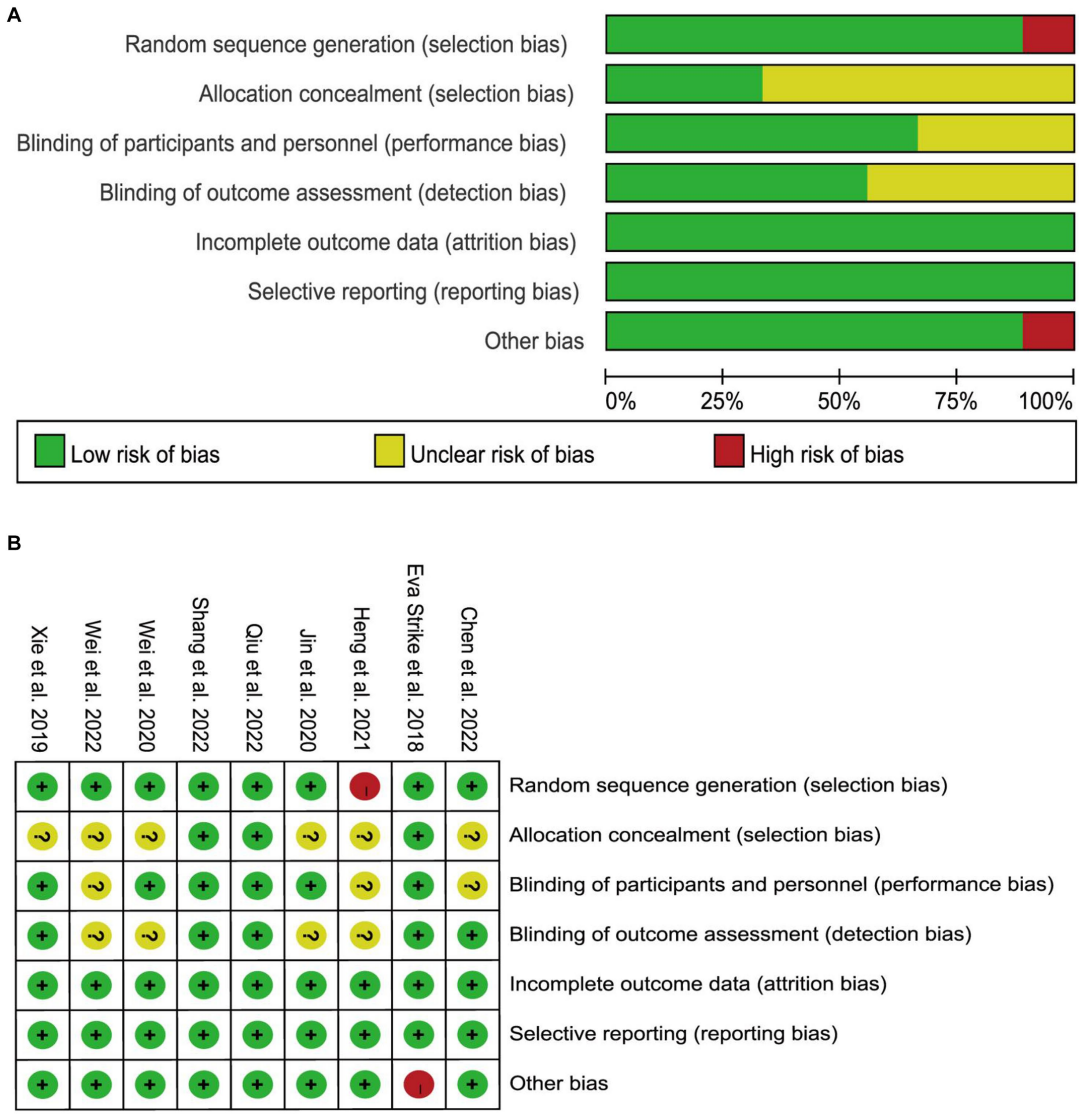
Risk of bias assessment for each study. **(A)** Risk of bias graph: review authors’ judgments about each risk of bias item presented as percentages across all included studies. **(B)** Risk of bias summary: review authors’ judgements about each risk of bias item for each included study.

Quality assessment results are presented in Figure 2. Six studies (Xie et al., 2019; Jin L. et al., 2020; Wei et al., 2020, 2022; Chen et al., 2022; Heng et al., 2022) failed to explicitly describe allocation concealment, while three studies (Chen et al., 2022; Heng et al., 2022; Wei et al., 2022) did not clearly state whether patients were blinded. Four (Jin L. et al., 2020; Wei et al., 2020, 2022; Heng et al., 2022) did not specifically indicate whether blinding was applied to those measuring cognitive outcomes. One study (Strike et al., 2019) was terminated early because due to clinical practice issues, as the authors were unable to include patients in a reasonable proportion of participants.

### Primary outcomes

#### Incidence of postoperative delirium, delayed neurocognitive recovery, and postoperative cognitive dysfunction

**Figure 3:**
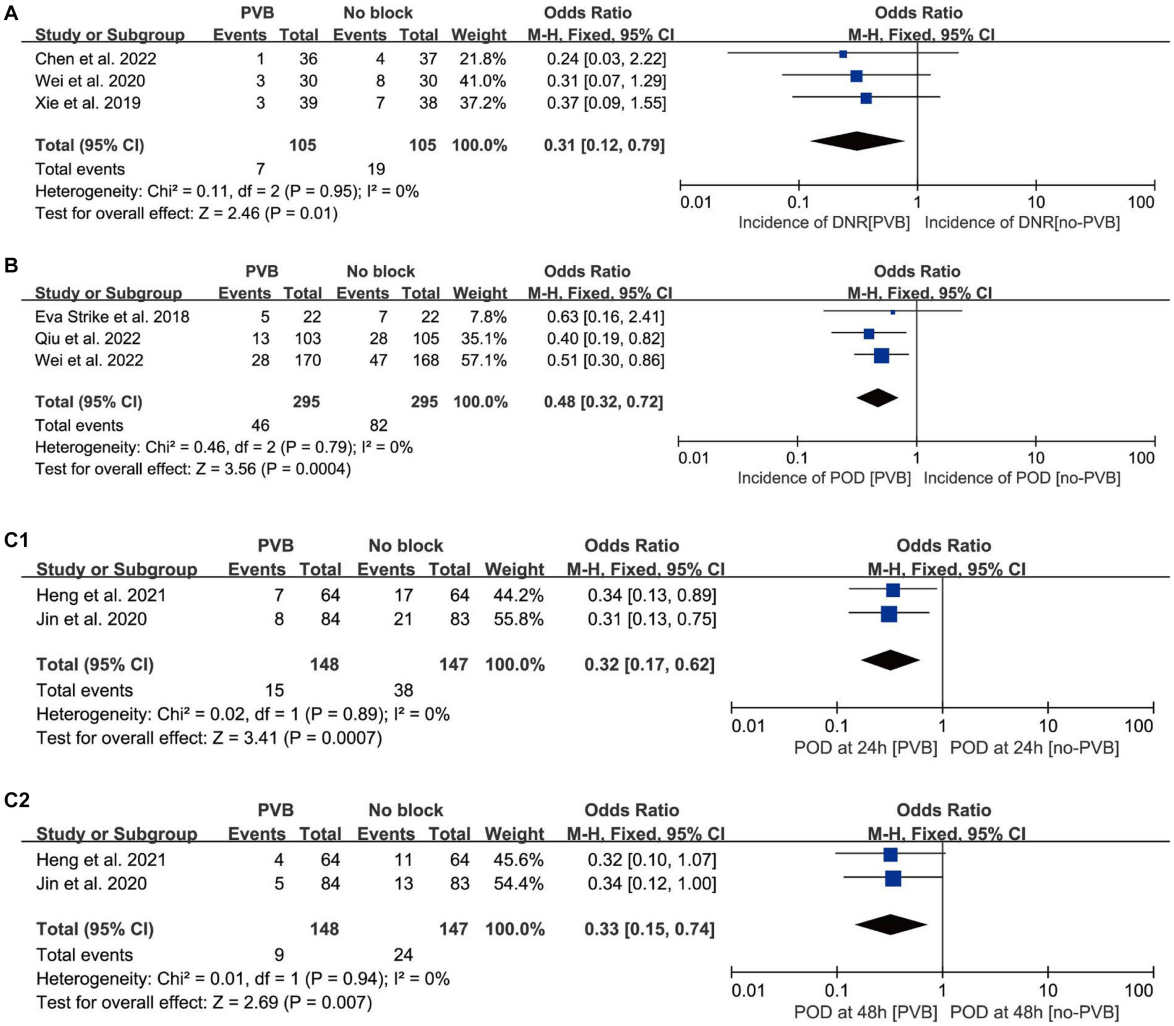
Forest plots showing pooled effect estimates for **(A)** the incidence of POCD; **(B)** the incidence of POD; **(C1)** the incidence of POD at 24 h; **(C2)** the incidence of POD at 48 h. PVB, Paravertebral block; POD, Postoperative delirium; POCD, Postoperative cognitive dysfunction.

Nine studies reported postoperative sedation or agitation levels and postoperative POD/DNR using different instruments (Table 3). The incidence of POD/DNR with and without the intervention of PVB are displayed in Figure 3. Three studies reported the occurrence of DNR 7 days after surgery (Xie et al., 2019; Wei et al., 2020; Chen et al., 2022) (Figure 3A), and PVB significantly reduced the incidence of DNR compared with non-PVB group [OR = 0.32, 95% CI 0.13, 0.80; *p* = 0.01; *I*^2^ = 0%], and Egger’s test showed no significant publication bias (*p* = 0.78). In addition, according to three studies, PVB also reduced POD incidence [OR = 0.48, 95% CI 0.32, 0.72; *p* = 0.0004; *I*^2^ = 0%] in patients who showed POD incidence after 7 days of surgery (Figure 3B). No significant (*p* = 0.86) publication bias was detected. Two studies reported the incidence of POD at 24 h (Figure 3C1) and 48 h (Figure 3C2) postoperatively (Jin L. et al., 2020; Heng et al., 2022). The results showed that PVB significantly reduced the incidence of POD at both 24 h [OR = 0.32, 95% CI 0.17, 0.62; *p* = 0.0007; *I*^2^ = 0%] and 48 h [OR = 0.33, 95% CI 0.15, 0.74; *p* = 0.007; *I*^2^ = 0%]. Egger’s test showed no significant publication bias (*p* = 0.894; *p* = 0.838). Only one study investigated POCD at 3 months postoperatively (Chen et al., 2022), and found that one patient (2.7%) in PVB group and four patients (10.8%) in non-PVB group experienced POCD. Sensitivity analysis confirmed the stability of model by using the one-by-one exclusion method.

**Table 3:** Cognitive assessment of included trials.

Research	Sedation or agitation assessment	Scale	POD assessment	Scale	Follow-up time point for POD	DNR assessment	Follow-up time point for DNR	Scale
Strike et al. (2019)	√	SAS (Every 4 h)	√	CAM-ICU	0–48 h	/	/	/
Xie et al. (2019)	/	/	/	/	/	√	7d	MMSE, digital symbol test, digital breadth test, trail connection test A
Jin Z. et al. (2020) and Jin L. et al. (2020)	√	RASS (the first 4d after surgery between 8:00–10:00 a.m.)	√	CAM	1d, 2d	/	/	/
Wei et al. (2020)	/	/	/	/	/	√	7d	MMSE
Heng et al. (2022)	√	RASS (the first 4d following surgery from 8:30–10:30 a.m.)	√	Nu-DESC	0–48 h	/	/	/
Wei et al. (2022)	/	/	√	3D-CAM	3d, 7d	/	/	/
Shang et al. (2022)	/	/	/	/	/	√	1d, 3d	MMSE
Chen et al. (2022)	√	RASS	/	/	/	√	1d	MMSE
Dongjie et al. (2023)	/	/	√	Nu-DESC	0–5d	/	/	/

SAS, Sedation-Agitation Scale; RASS, Richmond agitation sedation scale; Nu-DESC, The Nursing Delirium Screening Scale; MMSE, Mini-Mental State Examination; CAM, Confusion Assessment Method; 3D-CAM, 3-min Diagnostic Interview for CAM.

### Secondary outcomes

#### MMSE scores and S-100β levels

**Figure 4:**
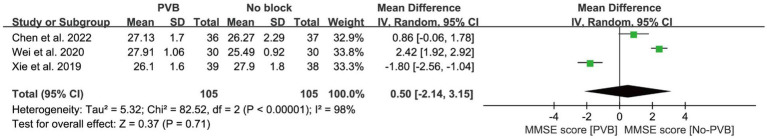
Forest plot: comparing MMSE score of patients receiving PVB vs. No-PVB.

**Figure 5:**
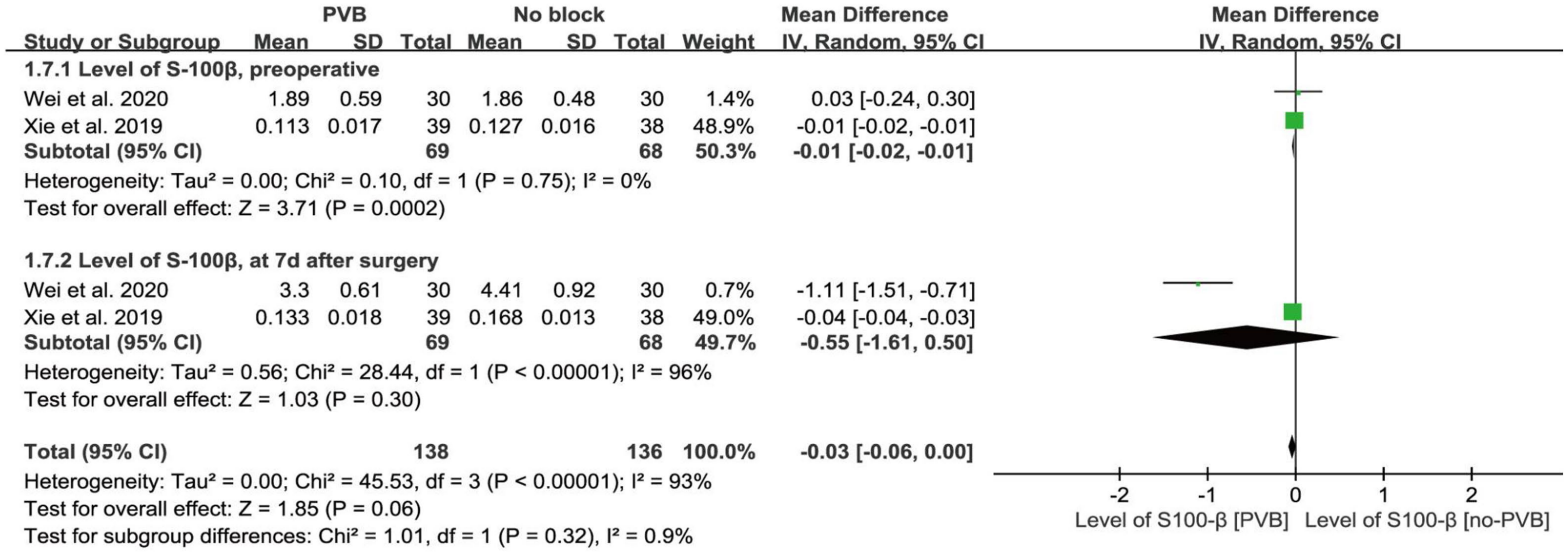
Forest plot: comparing plasma S-100β of patients receiving PVB vs. No-PVB.

Three studies (Xie et al., 2019; Wei et al., 2020; Chen et al., 2022) compared preoperative and postoperative MMSE scores, and the results showed no significant difference between the two groups [MD = 0.50, 95% CI −2.14, 3.15; *p* = 0.71; *I*^2^ = 98%] (Figure 4). Egger’s test did not detect significant publication bias (*p* = 0.069). Two RCTs (Xie et al., 2019; Wei et al., 2020) reported S-100β levels before surgery and on the seventh day after surgery (Figure 5), and there was no significant difference between the two groups after surgery [MD = −0.55, 95% CI −1.61, 0.50; *p* = 0.30; *I*^2^ = 96%]. Egger’s test showed no significant publication bias (*p* = 0.182).

#### Acute resting pain score

**Figure 6:**
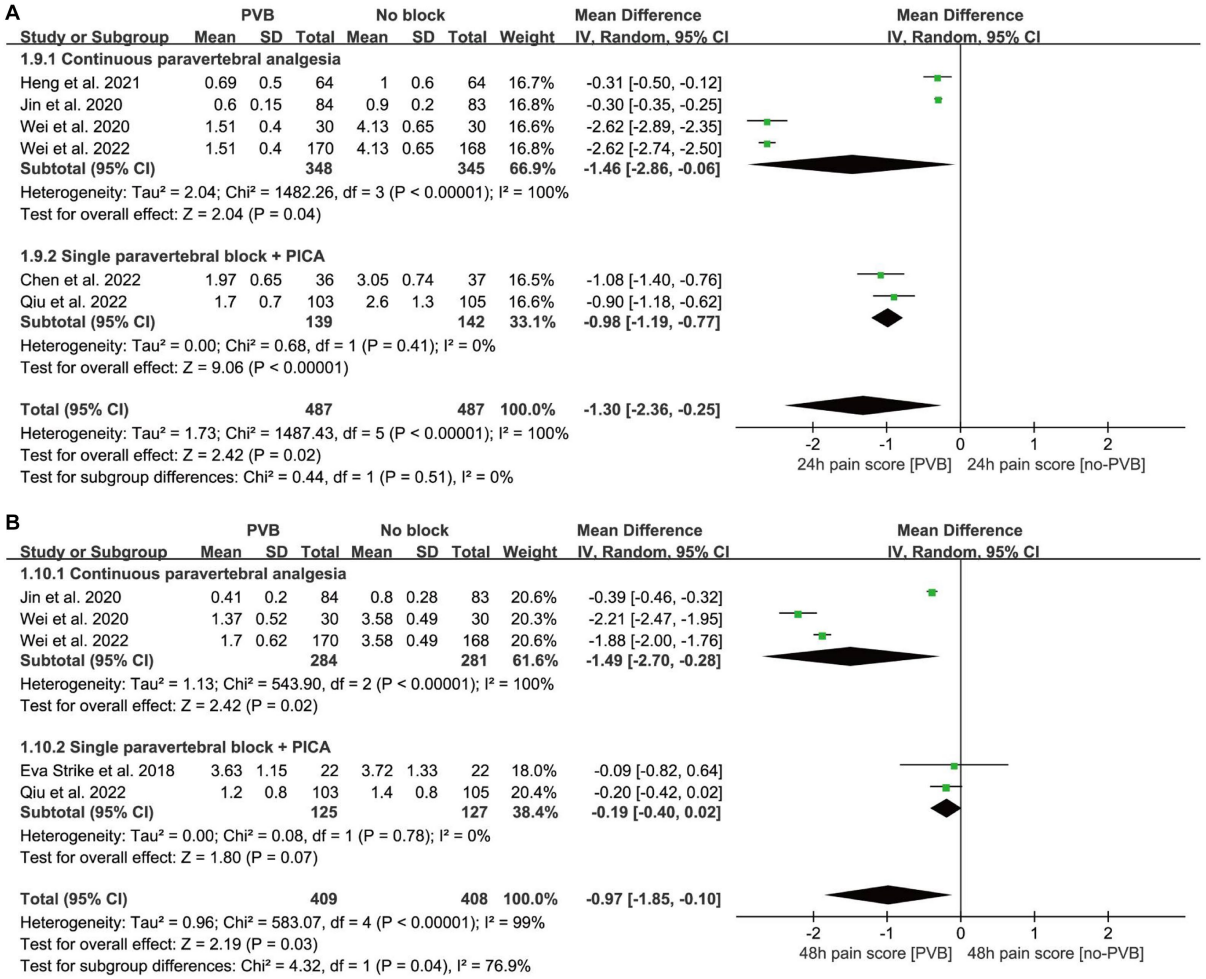
Forest plot: comparing postoperative pain score of patients receiving PVB vs. No-PVB. **(A)** Pain score at postoperative 24 h; **(B)** pain score at postoperative 48 h. PICA, patient-controlled intravenous analgesia; PVB, paravertebral block.

In total of 11 trials reported rest pain scores, six studies reported pain scores after 24 h (Jin L. et al., 2020; Wei et al., 2020, 2022; Chen et al., 2022; Heng et al., 2022; Dongjie et al., 2023) (Figure 6), while five studies (Strike et al., 2019; Jin L. et al., 2020; Wei et al., 2020, 2022; Dongjie et al., 2023) reported pain scores at 48 h. Further analysis revealed that PVB effectively reduced pain at 24 h [MD = −1.30, 95% CI −2.36, −0.25; *p* = 0.02; *I*^2^ = 100%] and 48 h [MD = −0.97, 95% CI −1.85, −0.10; *p* = 0.03; *I*^2^ = 99%] after surgery. Egger’s test identified no significant publication bias (*p* = 0.058; *p* = 0.266).

After 24 h of surgery, the combined results of the continuous paravertebral block (CPA) group (*p* = 0.04) and PVB + PCIA (patient-controlled intravenous analgesia) group (*p* < 0.01) were statistically significant, in agreement with the total combined results (*p* = 0.02, MD < 0), specifying that PVB had a protective effect on the prevention of PND at 24 h. Moreover, both PVB and CPA were independently effective. Although the total combined results (*p* = 0.03) and the results of CPA (*p* = 0.02) were statistically significant, single PVB + PCIA (*p* = 0.07) was not statistically significant at 48 h postoperative. Single PVB + PCIA and CPA had significant subgroup differences (*p* = 0.04) in the combined results.

#### Postoperative opioid consumption

**Figure 7:**
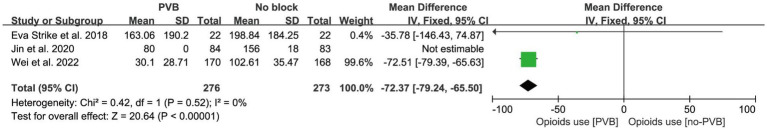
Forest plot: comparing opioids consumption (ug) of patients receiving PVB vs. No-PVB.

Three studies (Strike et al., 2019; Jin L. et al., 2020; Wei et al., 2022) reported opioid consumption for postoperative pain management. Meta-analysis was performed by converting the equivalent doses of various opioid drugs to sufentanil. All three studies applied continuous paravertebral analgesia with local anesthetics postoperatively (Table 1), supplemented with opioids, and showed that PVB significantly reduced postoperative opioid consumption [MD = −72.37, 95% CI −79.24, −65.50; *p* < 0.001; *I*^2^ = 0%] (Figure 7). Egger’s test showed no significant publication bias (*p* < 0.001).

#### Mean arterial pressure

**Figure 8:**
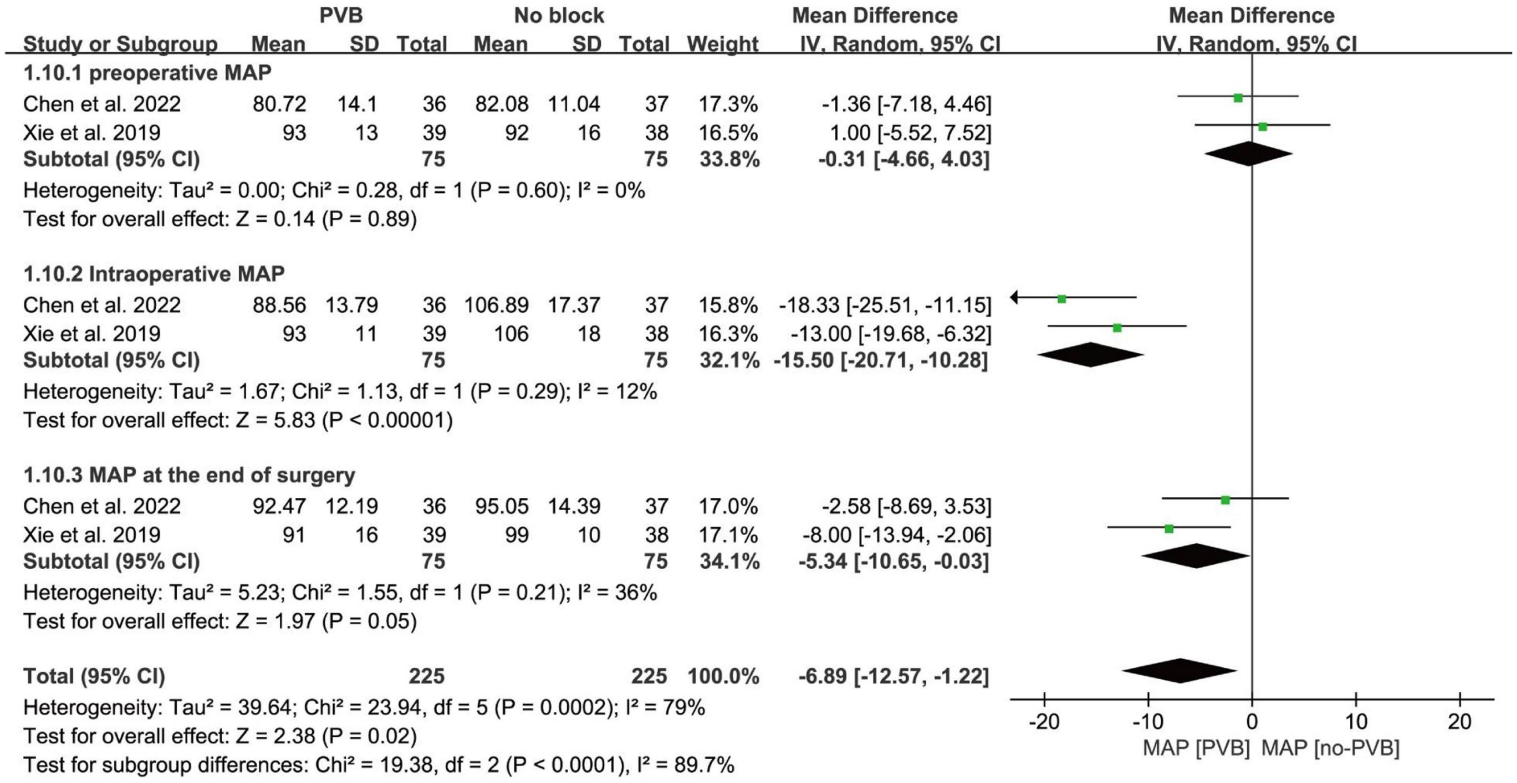
Forest plot: comparing MAP of patients receiving PVB vs. No-PVB. MAP: Mean Arterial Pressure.

Mean arterial pressure (MAP) was measured in patients preoperatively, intraoperatively and at the end of surgery in two studies (Xie et al., 2019; Chen et al., 2022), and there was no significant difference in MAP between the two groups preoperatively [MD = −0.31, 95% CI −4.66, 4.03; *p* = 0.89; *I*^2^ = 0%]. While the MAP measured intraoperatively [MD = −15.50, 95% CI −20.71, −10.28; *p* < 0.001; *I*^2^ = 12%] and postoperatively [MD = −5.34, 95% CI −10.65, −0.03 *p* = 0.05; *I*^2^ = 36%] was significantly lower in the PVB group than in the non-PVB group (Figure 8). Egger’s test did not show significant publication bias (*p* = 0.590; *p* = 0.401; *p* = 0.221).

#### Duration of hospitalization

**Figure 9:**
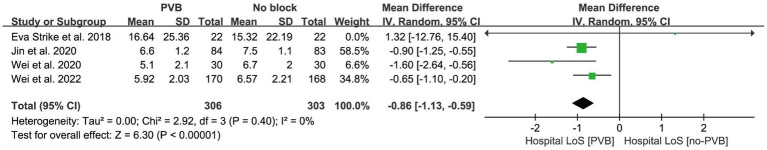
Forest plot: comparing hospital lengths of stay of patients receiving PVB vs. No-PVB. Hospital LoS: hospital lengths of stay.

Four studies included in the analysis reported length of hospital stay (Strike et al., 2019; Jin L. et al., 2020; Wei et al., 2020, 2022), the PVB group had a shorter hospital stay [MD = −0.86, 95% CI −1.13, −0.59; *p* < 0.001; *I*^2^ = 0%] (Figure 9). Egger’s test showed no significant publication bias (*p* = 0.688).

## Discussion

Postoperative delirium (POD), delayed neurocognitive recovery (DNR), and postoperative cognitive dysfunction (POCD) could be distinct manifestations of neurocognitive deficits, triggered by interactions between surgery, anesthesia and one or more preoperative vulnerabilities (e.g., inflammation, blood–brain barrier dysfunction). According to Daiello et al. (2019) POD can significantly increase the risk of DNR. Similar results were concluded by Huang et al. (2021) after meta-analysis. Recent years, studies have shown that peripheral nerve block (PNB) are potential to help lower the POD incidence. A Japanese retrospective, nationwide cohort study demonstrated that PNB combined with general analgesia was associated with a reduction of postoperative delirium (Yoshimura et al., 2022). Kim et al.’s (2023) meta-analysis also support that perioperative PNB can lower the POD incidence and pain scores up to the third postoperative day. The current meta-analysis suggests that PVB may be an effective component of multimodal analgesia to prevent perioperative neurocognitive disorder in patients undergoing major surgery.

This meta-analysis revealed that PVB significantly reduced the risk of POD, as confirmed by five trials (Strike et al., 2019; Jin L. et al., 2020; Heng et al., 2022; Wei et al., 2022; Dongjie et al., 2023). Among them, Heng et al. (2022) and Jin L. et al. (2020) investigated the incidence of delirium at 24 h and 48 h after surgery, respectively. A decrease in POD was observed with the passage of time and administration of PVB. In addition, the recovery of DNR was calculated based on the decrease of neurocognitive test scores compared with the preoperative scores. All three studies in our analysis used MMSE to measure neurocognitive function and showed that PVB helps reduce the risk of early DNR (Xie et al., 2019; Wei et al., 2020; Chen et al., 2022). Xie et al. (2019) found that PVB combined with general anesthesia (TPVB-GA) reduced postoperative cognitive impairment, as results were confirmed by five different parts including MMSE, digital symbol test, trail connection test A and digital breadth test. The other two studies showed an association between DNR and MMSE scores. Chen et al. (2022) showed that general anesthesia combined with PVB reduces DNR in patients undergoing thoracoscopic surgery. Similarly, Wei et al. (2020) found that general anesthesia combined with PVB significantly relieved postoperative neurocognitive disorders in elderly patients after thoracic surgery.

Several factors can influence postoperative neurocognitive outcomes such as surgery type, anesthesia management, perioperative hemodynamic stability, pain, sleep disturbances, and the burden of analgesic (e.g., opioids) and sedative medications. In our study, six RCTs reported postoperative acute pain scores (Strike et al., 2019; Jin L. et al., 2020; Wei et al., 2020, 2022; Chen et al., 2022; Heng et al., 2022; Dongjie et al., 2023). We observed a high degree of heterogeneity in the results for acute pain scores at both 24 h and 48 h of the CPA subgroup (*I*^2^ > 76%). The combined results of pain scores at 24 h remained unchanged upon sequential elimination of the studies (*p* > 0.05). Although in the study by Jin L. et al. (2020), patients in the experimental group received paravertebral catheterization and local anesthetics were given every 6 h until 48 h after surgery, PCIA analgesia was the main analgesia in both the experimental group and the control group. We speculate that this is the source of heterogeneity. Both single PVB + PCIA and CPA significantly reduced acute pain scores compared to PCIA 24 h after surgery. However, after 48 h of surgery, CPA was more effective in reducing pain scores compared to single PVB, potentially because single PVB has a limited duration of analgesia. Studies have shown that continuous paravertebral block is more effective in reducing pain 24 h after surgery than single PVB + PCIA or traditional intravenous analgesia (Liu et al., 2022; Singh et al., 2022). Moreover, PVB has been shown to reduce opioid consumption for postoperative pain relief and analgesic remedy (Strike et al., 2019; Jin L. et al., 2020; Wei et al., 2022). However, subgroup analysis of opioid consumption was not possible due to studies that exclusively used CPA. Both single PVB or continuous paravertebral analgesia reduced the use of opioids. Previous study has found that opioids can have negative effects on postoperative cognition (Warner et al., 2022), and limiting opioid use has been shown to reduce the incidence of PND. PVB inhibits sympathetic nerve activity, thereby reducing the stress response and systemic inflammation resulting from surgical trauma (Zhang et al., 2020). This helps maintain MAP at a relatively stable level under the effect of PVB (Pintaric et al., 2011; Zhen et al., 2022). Abnormal intraoperative cerebrovascular autoregulation was a potential risk factor for delirium after cardiac surgery (Chan and Aneman, 2019). Paravertebral analgesia helped maintain hemodynamic stability and ensured that the brain was protected from hypotension-related hypoperfusion, thereby reducing the occurrence of subclinical cerebral vascular events and associated negative cognitive events (Krzych et al., 2020). PVB has also been found to shorten the length of hospital stay and may have potential benefits for patients.

Although our analysis showed that PVB reduced the incidence of POD or early DNR, three studies (Wei et al., 2020) showed no significant difference in MMSE scores between PVB and non-PVB group. We observed a high degree of heterogeneity in the studies (*I*^2^ > 76%). However, upon sequential elimination of the studies, the combined results remained unchanged, with a significance level of *p* < 0.01. The discrepancy in the assessment time of the MMSE, with Chen et al. performing the test on the 1st day after surgery and Wei and Xie conducting it on the 7th day, might have contributed to the high heterogeneity. Besides, Wei et al. did not explicitly mention blinding of outcome assessment. The subgroup analyses were not feasible due to the inclusion of three articles in this review. In particular, Chen et al. (2022) found no significant difference in MMSE scores between PVB and control groups after 1 day of surgery. Whereas Xie et al. (2019) and Wei et al. (2020) found that MMSE scores were significantly higher in the PVB group than in the general anesthesia group at 7th day after surgery. Previous studies have identified that MMSE alone is not the best suitable tool for postoperative neurocognitive testing (Pinto et al., 2019; Jia et al., 2021) because it is not sensitive enough to small changes in postoperative neurocognition. Besides, age is a major risk factor for PND (Bramley et al., 2021), and young patients generally experience fewer neurocognitive disorders and recover more quickly than older patients. In the study carried by Chen et al.’s (2022), age of patients is between 45 and 65 years, that can be the reason of no significant difference in MMSE scores in PVB and non-PVB groups.

S-100β is a kind of calcium-binding protein mainly produced by astrocytes. Its release after surgery is associated with disruption of the blood brain barrier as part of the inflammatory response (Kapural et al., 2002; Mazzone et al., 2003). Though the level of S-100β have predictive values in surgery patients with PND, the correlation of S-100β with brain injury (particularly neuropsychiatric disturbances) is relatively weak (Lloyd et al., 2000; Whitaker et al., 2007). The combined results of two studies showed no significant difference in postoperative S-100β levels between the PVB and non-PVB groups. Difference in S-100β protein levels was recorded by Wei et al. (2020) after 7 days of surgery in PVB and general anesthesia group. The PVB group has less S-100β protein levels compared to general anesthesia group. Additionally, serum S-100β protein levels were lower in the PVB group than in the general anesthesia group at the time of surgical skin incision. An early pattern of S-100ß release appears during or shortly after surgery, which generally restores within 24 h (Yu et al., 2021). Biomarker concentrations decrease over time may explain the lack of changes observed in S-100β protein levels.

To our knowledge, this is the first meta-analysis to investigate the relationship between PVB and POD/DNR. The studies included were all randomized controlled trials and were of high quality, with careful monitoring and adjudication to ensure accuracy. Our study has some limitations that need to be addressed further. Firstly, lack of similar studies effects the rule out the possibility of potential residual confounding caused by unmeasured or unknown factors (e.g., age, type of surgery, duration of surgery, etc.). Secondly, most studies use MMSE, which has limitation in detecting PND. Additionally, only one study (Xie et al., 2019) used the ISCOPD-recommended neuropsychological test battery. Furthermore, follow-up was mostly limited to 7 days postoperatively, with only one study (Chen et al., 2022) exploring the PVB effect on patients’ cognitive function at 30 days and 3 months postoperatively. Lastly, the studies measured S-100β protein levels on the 7^th^ day after surgery, but this is weakly associated with PND and began to decline 24 h after surgery. Future research should explore more sensitive biomarkers for nerve injury and monitor changes in perioperative plasma concentrations of biomarkers.

## Conclusion

In conclusion, several randomized controlled trials have demonstrated the effectiveness of PVB in reducing the incidence of POD or early DNR in patients undergoing major surgery. Considering that different postoperative neurocognitive conditions represent different stages of decline in cognitive reserves, and cognition recovery following surgery holds critical implications for patients’ future quality of life. Therefore, further in-depth studies are needed to confirm the effect of PVB on both short-term and long-term postoperative neurocognition.

## Data availability statement

The original contributions presented in the study are included in the article/Supplementary material, further inquiries can be directed to the corresponding author.

## Author contributions

LW and FW presented the study design, data acquisition, and write the original draft. GG, TL, and BC performed literature review and revise the manuscript. LW and WK collected and performed formal data analysis. WL contributed to the interpretation of results and critically revise the manuscript. All authors contributed to the article and approved the submitted version.

## Footnotes

^1^https://www.statstodo.com/CombineMeansSDs.php
