# Supplementary material for: Impact of paravertebral block on perioperative neurocognitive disorder: a systematic review and meta-analysis of randomized controlled trials
Source: Front Aging Neurosci. 2023 Oct 3;15:1237001. doi: 10.3389/fnagi.2023.1237001 (PMC10580806; doi:10.3389/fnagi.2023.1237001)
Supplement: Supplementary file 1 [file Table_1.DOCX]

("thoracic paravertebral block"[Title/Abstract] OR "TPVB"[Title/Abstract] OR "PVB"[Title/Abstract] OR "paravertebral block"[Title/Abstract] OR "paravertebral analgesia"[Title/Abstract]) AND ("postoperative cognitive dysfunction"[Title/Abstract] OR "POCD"[Title/Abstract] OR "perioperative neurocognitive disorders"[Title/Abstract] OR "PND"[Title/Abstract] OR "delayed neurocognitive recovery"[Title/Abstract] OR "postoperative neurocognitive disorder"[Title/Abstract] OR "postoperative cognitive disorder"[Title/Abstract] OR "postoperative delirium"[Title/Abstract] OR "POD"[Title/Abstract])

The search dates ranged from the establishment of each database to April 2023.
